# Discovery and Genomic Characterization of a Novel Bat Sapovirus with Unusual Genomic Features and Phylogenetic Position

**DOI:** 10.1371/journal.pone.0034987

**Published:** 2012-04-13

**Authors:** Herman Tse, Wan-Mui Chan, Kenneth S. M. Li, Susanna K. P. Lau, Patrick C. Y. Woo, Kwok-Yung Yuen

**Affiliations:** 1 Department of Microbiology, The University of Hong Kong, Hong Kong, China; 2 Research Centre of Infection and Immunity, The University of Hong Kong, Hong Kong, China; 3 State Key Laboratory of Emerging Infectious Diseases, Hong Kong Special Administrative Region, Hong Kong SAR, China; Institut Pasteur, France

## Abstract

*Sapovirus* is a genus of caliciviruses that are known to cause enteric disease in humans and animals. There is considerable genetic diversity among the sapoviruses, which are classified into different genogroups based on phylogenetic analysis of the full-length capsid protein sequence. While several mammalian species, including humans, pigs, minks, and dogs, have been identified as animal hosts for sapoviruses, there were no reports of sapoviruses in bats in spite of their biological diversity. In this report, we present the results of a targeted surveillance study in different bat species in Hong Kong. Five of the 321 specimens from the bat species, *Hipposideros pomona*, were found to be positive for sapoviruses by RT-PCR. Complete or nearly full-length genome sequences of approximately 7.7 kb in length were obtained for three strains, which showed similar organization of the genome compared to other sapoviruses. Interestingly, they possess many genomic features atypical of most sapoviruses, like high G+C content and minimal CpG suppression. Phylogenetic analysis of the viral proteins suggested that the bat sapovirus descended from an ancestral sapovirus lineage and is most closely related to the porcine sapoviruses. Codon usage analysis showed that the bat sapovirus genome has greater codon usage bias relative to other sapovirus genomes. In summary, we report the discovery and genomic characterization of the first bat calicivirus, which appears to have evolved under different conditions after early divergence from other sapovirus lineages.

## Introduction

The caliciviruses are a family of small non-enveloped viruses, and can be classified into five genera: *Vesivirus*, *Lagovirus*, *Norovirus*, *Sapovirus* and *Nebovirus*. They possess a non-segmented, polyadenylated, positive-sense ssRNA genome of about 7.5 to 8.5 kb in length, enclosed in an icosahedral capsid of 27 to 40 nm in diameter. Among them, noroviruses and sapoviruses (SaVs) are well known to cause enteric disease in a range of mammals, including humans, while vesiviruses and lagoviruses cause systemic diseases in specific animal hosts. *Nebovirus* is the most recently established genus in the family *Caliciviridae* [1], and its members are associated with enteric diseases in cattle [2], [3]. A putative sixth genus, *Recovirus*, has been proposed for a novel calicivirus detected in stool specimens from rhesus monkeys [4], [5]. Another new genus, *Valovirus*, has been proposed for a novel group of swine caliciviruses known as the St-Valérien-like viruses [6]. In addition, there exist other unclassified caliciviruses, such as the recently described chicken calicivirus [7].

The genus *Sapovirus* currently contains only one recognized species, the Sapporo virus, which was discovered in 1977 in Sapporo, Japan [8]. The SaV genome is approximately 7.1 to 7.5 kb in length, and may have two or three ORFs. ORF1 encodes a polyprotein that undergoes proteolytic cleavage to form the non-structural proteins and the major capsid protein VP1. ORF2 encodes the minor structural protein VP2. ORF3 encodes a small basic protein of unknown function [9], [10]. Interestingly, it is located in an overlapping reading frame within ORF1, and is present only in SaVs from selected genogroups. At present, SaVs are classified formally into 5 genogroups based on phylogenetic analysis of the full-length VP1 sequence, though additional genogroups have been proposed to accommodate some novel SaVs discovered in recent years. Further classification of SaVs into genotypes has also been undertaken, though taxonomic assignment at the genotype level appears to be less well-defined than at the genogroup level [11].

As mentioned above, both noroviruses and SaVs generally cause mild to asymptomatic enteric infections in human and animal hosts [12]. Human SaV infections are reported to be similar to or milder than human norovirus infections, but SaV infections have a shorter duration of viral shedding and are less associated with projectile vomiting [13]–[16]. Incidence of SaV-associated gastroenteritis infections remains less than norovirus-associated infections for both sporadic and outbreak settings, though various studies have reported increasing rates of SaV infections around the world [17]–[21]. The genetic diversity of SaVs is comparable to that of noroviruses, and the diversity of reported animal hosts is also similar. Noroviruses have been discovered in specimens from humans, pigs, cattle, dogs, sea lions, African lion, and mice [22]–[25]. In comparison, SaVs have been found in specimens from humans, pigs, dogs, minks and California sea lions [23], [25]–[27].

Bats (order Chiroptera of class Mammalia) constitute a significant portion of biological diversity in many ecosystems and have a wide geographical distribution [28]. We have previously discovered novel viruses in several local bat species [29]–[35], and there were many similar discoveries of novel bat viruses by researchers in other parts of the world. In particular, important human viral pathogens like the SARS virus, Nipah virus and Ebola virus were found to have originated from bats and contributed to substantial human morbidity and mortality in recent outbreaks. Taken together, these discoveries hint that these small mammals are important reservoirs of diverse and undiscovered animal viruses, with significant risk of zoonotic transmission to humans [36].

In the present study, we investigated the presence of unknown calicivirus diversity in bats by targeted RT-PCR screening. Novel SaV sequences were amplified from several faecal samples of the bat species *Hipposideros pomona*, and genome sequences were obtained for three strains of the bat SaV. Sequence analysis indicated that the novel virus possesses several genomic features atypical of SaVs, and phylogenetic analysis revealed that it descended from a lineage that had diverged early from other SaV.

## Results

### Surveillance and detection of novel SaVs in bats

A total of 728 anal swabs from different bat species in Hong Kong were obtained. No obvious signs of enteric disease, like anorexia and diarrhoea, were observed in the bats during the brief period of captivity needed for sampling.

RT-PCR using broadly reactive degenerate primers for a 185 nt fragment in the 3D-like RNA-dependent RNA polymerase (RdRp) region of the calicivirus ORF1 gene was positive in two specimens. Repeated screening using more sensitive specific primers revealed three additional positive specimens. Further information on the species and RT-PCR screening results are presented in Table 1, Table S1 and Figure S1. Sequence similarity search using BLASTN against the NCBI non-redundant nucleotide database did not reveal significant similarity to known SaV sequences. Another search using BLASTX against the NCBI non-redundant protein database produced hits to SaV sequences, with the most closely related sequence being the RdRp sequence of porcine SaV (GenBank accession number ACT98315) at 43% aa identity. A phylogenetic tree was constructed from the nucleotide alignment based on the length of the partial RdRp sequence obtained from bat SaV/TLC72 (GenBank accession number JQ267527) (Figure S2).

**Table 1:** Species distribution of specimens and RT-PCR surveillance results in the present study.

Bat species	Number of animals screened by RT-PCR	Number (%) of animals with positive detection of SaV
*Hipposideros armiger*	14	-
*Myotis ricketti*	103	-
*Miniopterus pusillus*	78	-
*Myotis chinensis*	18	-
*Rhinolophus sinicus*	65	-
*Tylonycteris pachypus*	14	-
*Hipposideros pomona*	321	5 (1.56%)
*Pipistrellus abramus*	9	-
*Miniopterus schreibersii*	84	-
*Nyctalus noctula*	1	-
*Pipistrellus* sp.	2	-
*Scotophilus kuhlii*	1	-
*Rhinolophus affinis*	9	-
*Rhinolophus pusillus*	9	-

### Genome sequencing and analysis of novel bat SaVs

**Figure 1:**
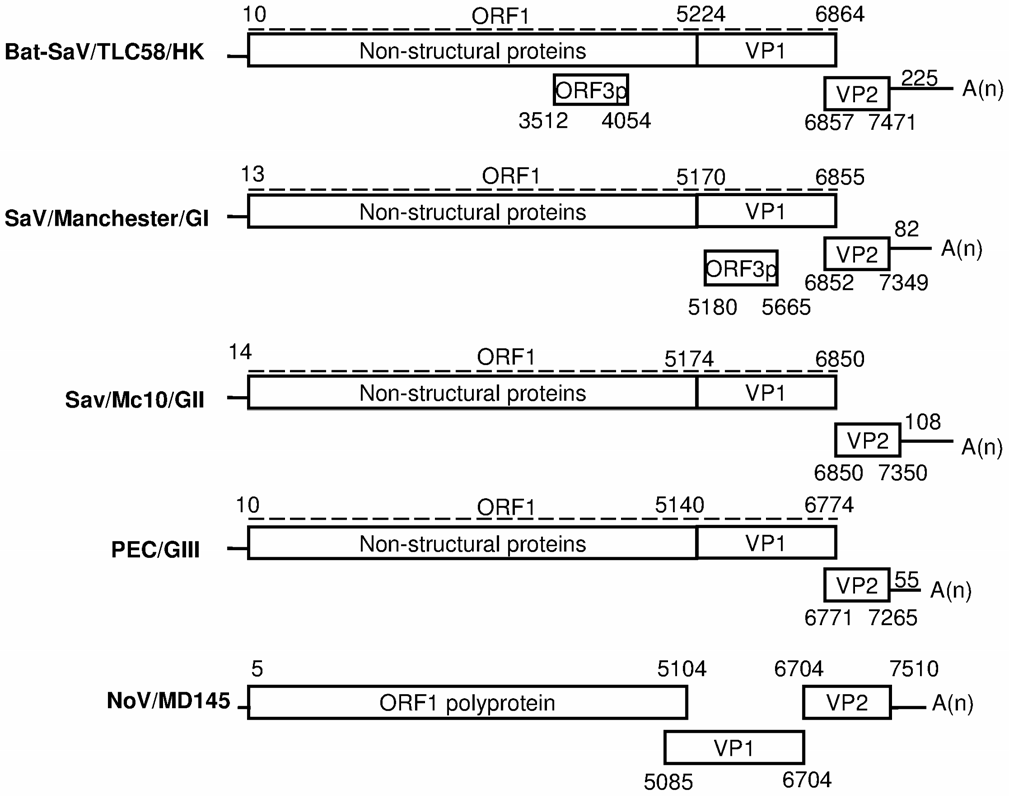
Genome organization of the bat SaV TLC58/HK. The genome organization of the bat SaV TLC58/HK in comparison with the genome organization of human SaV GI strain Mancheseter, human SaV GII strain Mc10, porcine enteric calicivirus, and norovirus GII strain MD145.

Complete or nearly full-length genome sequences (with incomplete 5′ ends) were obtained for three positive samples using the sequencing strategy as described in the Methods section. For two of the samples that were positive only for RT-PCR screening with specific primers, only sequences for short segments of the viral genome were obtained. Additional viral genome sequencing on these samples was unsuccessful due to limited clinical materials available and possibly low viral titres. The complete genome of bat SaV strain TLC58 (Genbank accession number JN899075) is 7696 nt in length and has a genomic G+C content of 60.2 mol%. Both the length and G+C content of the bat SaV genome are significantly higher than that of other known SaVs (Table 2). Each genome is predicted to contain 3 overlapping ORFs, comparable to the genome organization of SaVs in GI, GIV and GV (Figure 1). The 5′-UTR and the 3′-UTR are 9 nt and 225 nt in length, respectively. The length of the 3′-UTR is considerably longer than other SaVs (Table 2). The two other nearly full-length bat SaV genomes were found to be highly similar to that of the complete bat SaV/TLC58 genome in nucleotide sequence and genome organization, and were not analysed separately.

**Table 2:** Comparison of genomic features among selected SaV.

Virus	Genogroup*	Genome length (nt)	Genomic G+C content (mol%)	ORF1 length (nt)	ORF2 length (nt)	3′-UTR length (nt)	ORF2 reading frame relative to ORF1	Presence of putative ORF3
Bat SaV/TLC58/HK	N/A	7696	60.2	6855	615	225	+1	Y
SaV/Manchester	I	7431	50.7	6843	498	82	−1	Y
SaV/Mc10	II	7548	51.5	6837	501	108	−1	N
Porcine enteric calicivirus	III	7320	53.5	6765	495	55	−1	N
SaV/Hu/Chiba/000671/1999/JP	IV	7420	53.5	6816	504	91	−1	Y
SaV/Hu/Ehime475/2004/JP	V	7500	49.8	6906	501	83	−1	Y
Canine SaV†	N/A	Incomplete	Incomplete	Incomplete	501	141	+1	N
California sea lion 1 SaV†	N/A	Incomplete	Incomplete	Incomplete	504	Incomplete	−1	N

* Only the formally recognized SaV genogroups are included.

† Complete genome sequence is not available for canine SaV and California sea lion 1 SaV.

Sequence accession numbers are as follows: Bat SaV/TLC58/HK (JN899075), SaV/Manchester (X86560), SaV/Mc10 (NC_010624), porcine enteric calicivirus (PEC) (AF182760), SaV/Hu/Chiba/000671/1999/JP (AJ786349), SaV/Hu/Ehime475/2004/JP (DQ366344), canine SaV (JN387134), California sea lion 1 SaV (JN420370).

The complete ORF1 is 6855 nt long, and encodes a large precursor polyprotein with an estimated molecular mass of 246.8 kDa. The polyprotein contains characteristic amino acid motifs conserved in caliciviruses: 2C-like NTPase at residue 482 (GPPGIGKT), VPg at residues 958 (KGKTK) and 972 (SEYEE), 3C-like protease at residue 1183 (GDCG), 3D-like RNA-dependent RNA polymerase at residues 1520 (GLPSG) and 1568 (YGDD), and VP1 at residue 1867 (PPG). It undergoes proteolytic processing to produce the nonstructural viral proteins and the major capsid protein VP1. Based on comparison with the ORF1 cleavage map of SaV/Mc10 [37], [38], a human SaV GII strain, we predicted the cleavage site that generates the major capsid protein to be located between residues 1740 (E) and 1741 (G). An in-frame AUG start codon is located in a favourable context for translation initiation (GUGUUUGUGAUGGA) just upstream to the cleavage site, which has also been reported in other caliciviruses [6], [39]. This sequence is noted to be similar to the 5′ UTR of the genome, and it was postulated that the site might permit internal translation initiation from subgenomic RNA [39]. The sequence identities of the bat SaV/TLC58 with other SaVs in the complete ORF1 protein sequence vary between 36.0% and 37.4% (Table 3). While comparison with caliciviruses of other genera, the ORF1 sequence identities are overall lower (15.6%–22.8%) than those between different SaVs (Table S2). For individual alignment of protease – polymerase, sequence identities with other SaVs (45.3%–48.4%) are overall higher than those with other genera (22.7%–32.1%) (Table 3 and Table S2). The VP1 is predicted to be 546 aa long, and has a molecular mass of 56.6 kDa. It shares 36.1% to 39.2% amino acid identities with VP1 of other SaVs (Table 3). Likewise analysis with caliciviruses of other genera reveals lower similarities with 14.9% to 23.4% sequence identities (Table S2).

**Table 3:** Comparison of genome identities and amino acid identities between the predicted polyproteins of bat SaV and the selected SaV.

Viruses	Genogroup	Bat SaV/TLC34/HK					Bat SaV/TLC39/HK					Bat SaV/TLC58/HK				
		Pairwise nt identity (%)	Pairwise amino acid identity (%)				Pairwise nt identity (%)	Pairwise amino acid identity (%)				Pairwise nt identity (%)	Pairwise amino acid identity (%)			
		Complete genome	ORF1	Pro-Pol	VP1	VP2	Complete genome	ORF1	Pro-Pol	VP1	VP2	Complete genome	ORF1	Pro-Pol	VP1	VP2
SaV/Manchester	GI	44.7	36.3	47.2	36.3	16.9	44.8	36.4	47.5	35.8	16.9	44.8	36.4	47.5	36.1	16.9
SaV/Mc10	GII	45.2	37.1	48.1	37.5	17.4	45.2	37.1	48.2	37.5	17.4	45.2	37.1	48.2	37.3	17.4
PEC	GIII	45.3	36.5	45.4	36.3	15.5	45.3	36.7	45.7	36.6	15.5	45.3	36.6	45.7	36.3	15.5
SaV/Chiba/000671	GIV	46.1	37.3	48.2	38.5	19.9	46.0	37.4	48.4	38.2	19.9	46.0	37.4	48.4	38.3	19.9
SaV/Ehime475	GV	44.2	35.9	45.9	36.9	19.4	44.2	36.0	46.1	37.1	19.4	44.2	36.0	46.1	36.9	19.4
Canine SaV	N/A	N/A	N/A	46.7	39.3	16.5	N/A	N/A	47.0	39.2	16.5	N/A	N/A	47.0	39.2	16.5
California sea lion 1 SaV	N/A	N/A	N/A	45.2	38.9	17.9	N/A	N/A	45.3	38.9	17.9	N/A	N/A	45.3	38.9	17.9
Bat SaV/TLC34/HK	N/A	-	-	-	-	-	99.5	99.3	99.7	98.5	100	99.6	99.6	99.7	99.2	100
Bat SaV/TLC39/HK	N/A	99.5	99.3	99.7	98.5	100	-	-	-	-	-	99.8	99.6	100	99.0	100
Bat SaV/TLC58/HK	N/A	99.6	99.6	99.7	99.2	100	99.8	99.6	100	99.0	100	-	-	-	-	-

Pro-Pol: Protease - Polymerase.

The complete ORF2 is 615 nt long, with an overlapping region of 8 nt with the 3′ terminus of ORF1. Its reading frame is +1 relative to that of ORF1, unlike most other SaVs (Table 2). ORF2 encodes the minor structural protein VP2, which has an estimated molecular mass of 21 kDa. The mechanism of translation initiation in ORF2 of SaVs has not been fully elucidated. In the present case, a translational upstream ribosome binding site (TURBS) motif (CAUGGGACC; underline indicates region complementary to 18 S ribosomal rRNA sequence) could be identified at 24 nt upstream of the ORF2 start codon. Sequence identities for VP2 with other SaVs vary from 15.5% to 19.9% (Table 3). By comparison with caliciviruses of other genera, sequence identities are generally lower than those between SaVs. (4.8%–12.3%) (Table S2).

### Phylogenetic and recombination analysis

**Figure 2:**
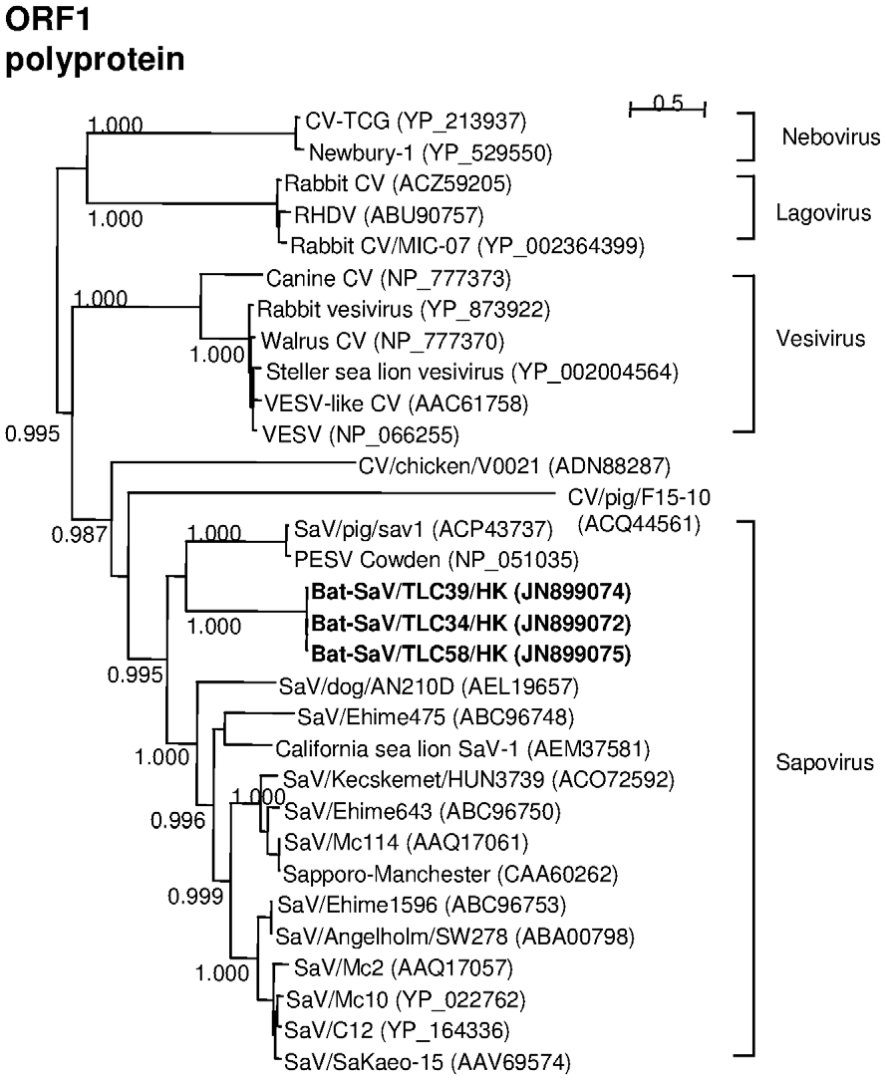
Unrooted maximum-likelihood tree based on full-length amino acid sequences of ORF1 precusor polyprotein. SH-like aLRT branch support values of greater than 0.70 are shown besides major branches. Scale bar indicates the number of inferred substitutions per site.

**Figure 3:**
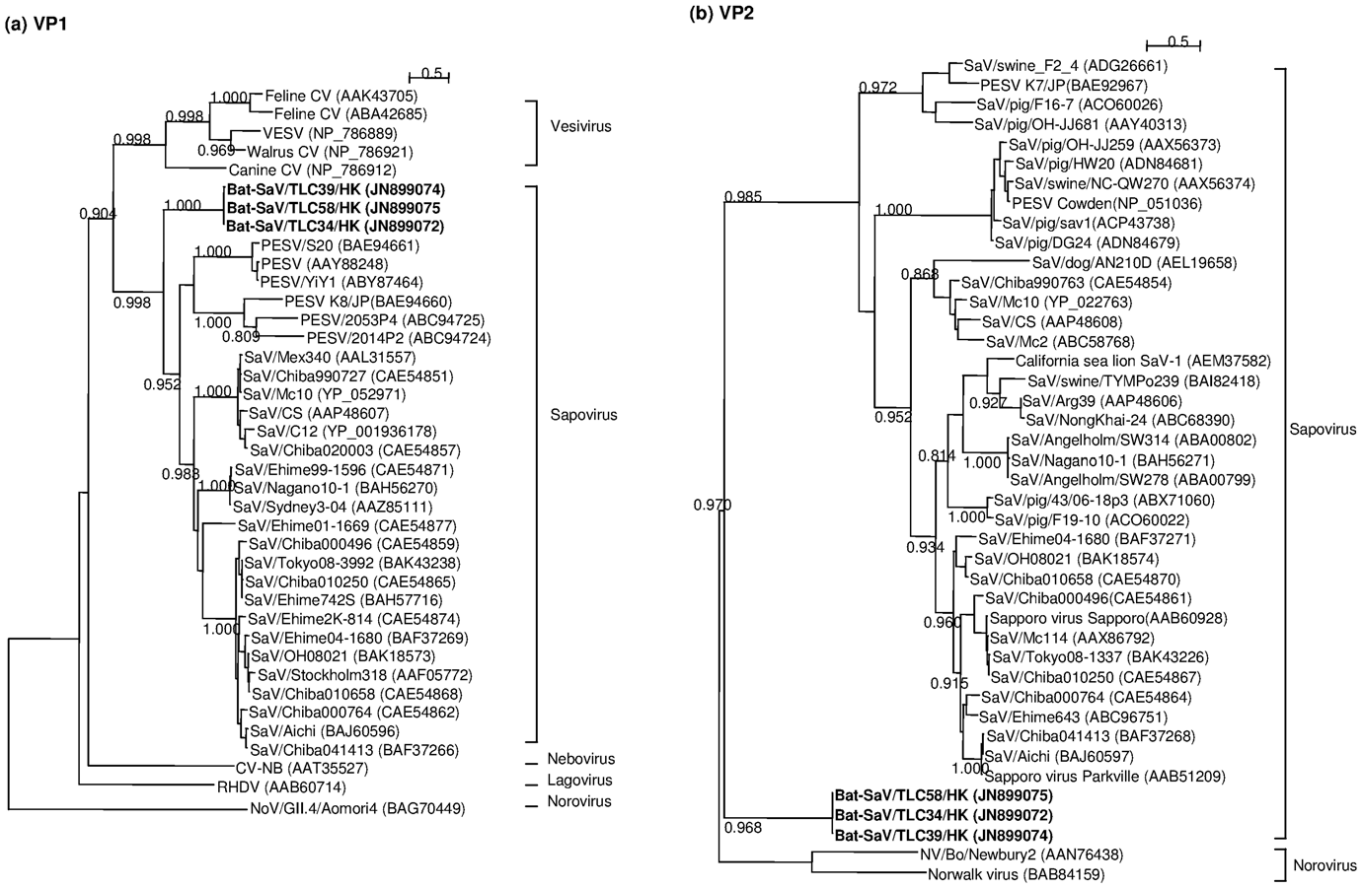
Unrooted maximum-likelihood trees of VP1 and VP2. The trees were constructed based on the full-length amino acid sequences of (a) VP1 major capsid protein, and (b) VP2 minor structural protein. SH-like aLRT branch support values of greater than 0.70 are shown besides major branches. Scale bar indicates the number of inferred substitutions per site.

Phylogenetic trees were constructed using the predicted amino acid sequences of the ORF1 precursor polyprotein (Figure 2), VP1 and VP2 (Figure 3). The LG+G+F model was found to be the best-fit substitution model in all cases. Phylogenetic analysis was not performed for the putative ORF3 product as no homologous sequences were available. Sequence analysis with the Recombination Analysis Tool did not reveal any potential recombination breakpoints in the bat SaV sequences.

There are subtle but important differences in the phylogenetic position of the bat SaV in the three phylogenetic trees. In the tree based on the full-length amino acid sequences of the ORF1 polyprotein, the bat SaVs are clustered tightly with the porcine SaVs in a monophyletic clade constituting the SaVs. However, in the VP1 tree, the bat SaVs are positioned just outside the clade of other SaVs. In the VP2 tree, the bat SaVs are located approximately equidistant from the GII noroviruses and porcine SaVs. The phylogenetic positions of the bat SaVs are supported by high Shimodaira-Hasegawa-like approximate likelihood ratio test (SH-like aLRT) branch support values as calculated by PhyML.

Although the phylogenetic positions of the novel bat virus are slightly divergent in the three trees, they generally show the bat SaV as being most closely related to the SaVs. In our opinion, there is insufficient ground for proposing a new genus for the novel virus under the current framework of taxonomic classification. The ORF1 polyprotein and VP1 capsid protein sequences of the novel bat virus showed obvious phylogenetic clustering with other SaV sequences. It should also be noted that the VP2 protein sequences are shorter and more divergent, and therefore are considered to be less useful in the phylogenetic classification of caliciviruses [4]. Lastly, the genome organization of the bat SaV is highly similar to that of the SaVs as shown above. Hence, together with relatively high sequence identities with other SaVs rather than with calicivirues in other genera (Table S2), we propose that the novel bat virus be classified as a new member of the genus *Sapovirus* in the family *Caliciviridae*.

### Codon usage and compositional bias analysis

**Figure 4:**
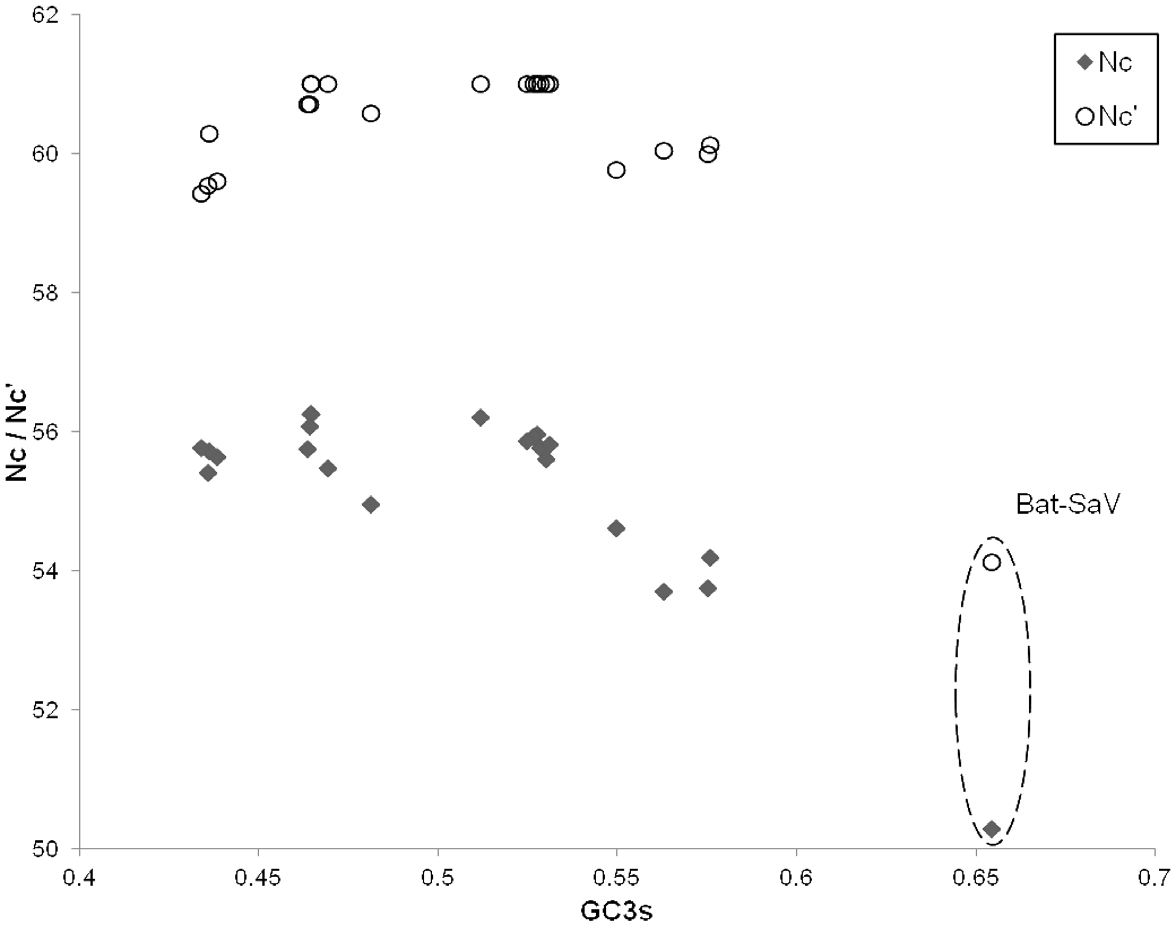
Scatterplot of codon usage. The scratterplot of codon usage summary statistics N_c_ and N_c_′ against the proportion of G or C nucleotides at the 3^rd^ position of synonymous codons (GC_3s_), showing greater codon usage bias in the bat SaV genome relative to other SaV genomes. Unlike the porcine enteric caliciviruses, the observed difference in codon usage bias persists with adjustment of background nucleotide composition (N_c_′).

As genomic nucleotide composition is strongly associated with codon usage bias in viruses, we examined the codon usage in the genomes of the novel bat SaV and other SaVs given their different nucleotide composition. The bat SaV genome was found to have significantly greater codon usage bias than the other SaV genomes, as measured by their effective number of codons (N_c_) (Figure 4). Adjusting N_c_ for background nucleotide composition (N_c_′) did not significantly affect the observed difference in codon usage bias.

Next, we examined CpG dinucleotide bias in the SaV genomes, as studies on other animal RNA viruses suggest that CpG suppression is a major factor in their genome evolution [40], [41]. Odds ratio of CpG and GpC dinucleotides (ρ_CG_ and ρ_GC_) and the CpG/GpC ratio were calculated to assess the degree of CpG suppression. Results confirm the presence of significant CpG suppression (ρ_CG_≤0.78) in examined SaV genomes, with the only exception being the bat SaV genome (Table 4). ρ_GC_ values are similar across examined SaV genomes, suggesting that the difference in CpG suppression is specific. All SaV genomes are found to have a slightly negative GC skew, and there is no major difference between the degree of GC skew in bat SaV and the other SaVs (Table 4). This suggests that the degree of cytosine deamination is not a major factor in the altered GC composition and CpG suppression in the bat SaV genome.

**Table 4:** CpG dinucleotide bias in selected SaV genomes, as assessed by the odds ratio of CpG (ρ_CG_) and other measures.

Virus	SaV genogroup	ρ_CG_	ρ_GC_	CpG/GpC ratio	GC skew
Bat SaV/TLC58/HK	N/A	0.792	1.017	0.778	−0.011
SaV/Manchester	I	0.530	0.954	0.556	−0.017
SaV/Mc10	II	0.587	0.938	0.626	−0.032
Porcine enteric calicivirus	III	0.520	1.016	0.511	−0.016
SaV/Hu/Chiba/000671/1999/JP	IV	0.665	0.976	0.681	−0.045
SaV/Hu/Ehime475/2004/JP	V	0.490	0.968	0.507	−0.036

Significantly less CpG suppression was found in the bat SaV genome, while a similar degree of negative GC skew was observed in all SaV genomes.

## Discussion

Although the taxonomic classification of caliciviruses has improved with the availability of full-length gene sequences and robust phylogenetic methods [42], the increase in genetic diversity introduced by novel caliciviruses would necessitate further taxonomic revisions within the family. The International Committee on Taxonomy of Viruses has adopted a systematic polythetic approach towards virus taxonomy, but classification at or below the genus level may be complicated by the specific biology of diverse viruses. As a case in point, the proposed assignment of the novel bat virus to the genus *Sapovirus* might be opposed on the basis of an increased genomic G+C content, the different reading frame of ORF2, and the increased length of the 3′ UTR. On the other hand, the polythetic criteria for inclusion in the genus are not fully clear, and phylogenetic distances between viral gene sequences have assumed overriding importance in previous and current classifications. It should be noted that even phylogenetic analysis may be confounded by other factors such as the cleavage pattern of ORF1 polyprotein, which has not been determined experimentally for many caliciviruses.

Among the various notable genomic features and properties in the novel bat SaV, we were most intrigued by its remarkably high G+C genomic content. Most caliciviruses have a genomic G+C content of 44.2–57.4 mol%. Among them, the genomic G+C content of the SaVs lie within the relatively narrow range of 49.0–53.6 mol% in spite of their genetic diversity. Hence, the presently observed G+C genomic content of 60.2% is significantly higher than that for other SaVs or caliciviruses, and indeed would rank amongst mammalian RNA viruses with the highest G+C genomic content [43]. Relatively little is known about the evolution of genome composition in caliciviruses. A number of factors have been postulated to exert selectional pressure on the G+C content of viral genomes, including host body temperature, immune pressure, codon and nucleotide usage patterns [44]–[47]. Our results suggested that the increased G+C content is associated with a decrease in CpG suppression, but does not have a direct correlation with codon usage bias. We are unaware of any previous findings indicating that genomes of bat viruses are under less CpG suppression, thus the observed reduction in CpG suppresion is unlikely to result from host-related factors. The greater codon usage bias in the bat SaV genome is another interesting genome feature, which could be associated with altered dinucleotide frequencies. The association could be tested by Markov modelling of the dinucleotide and codon frequencies in the SaV genomes, although the small genome sizes and the presently small number of complete genomes would limit the usefulness of this approach [48].

The novel SaV described presently is the first known member of the *Caliciviridae* in bats. The approach to its discovery is based on the established strategy of targeted genetic screening informed by conserved sequences of related viruses. Although this “homology-based” strategy has been successful in the discovery of numerous viruses, the advent of affordable high-density microarrays and high-thoughput sequencing has given rise to virus discovery through metagenomics. Indeed, the first canine SaVs were discovered recently by metagenome sequencing of canine diarrhoea samples on a high-throughput pyrosequencer [23]. Important advantages of the new method include detection of novel viruses not closely related to known viruses, and the capacity to detect multiple divergent viruses in cases of co-infection. However, metagenomics sequencing can suffer from possible bias during sample preparation [49], and it is unlikely to detect very low titres of viruses in a specimen, such as the three bat faecal samples that were positive upon repeat screening with specific PCR primers in the present study. While we anticipate the increasing utilization of the metagenomics approach, existing methods such as viral culture, electron microscopy and targeted nucleic acid amplification would continue to serve important roles in virus discovery.

As Hong Kong is a highly urbanized city, the local roosting sites of bats are mainly man-made structures, such as water tunnels and abandoned mines. *Hipposideros pomona* is very common and widespread throughout Hong Kong countryside areas. It is a small-sized leaf-nosed bat with body weight ranged from 6–8 g. It possesses a small nose leaf which is simple, small, and lacking of lateral leaflets (Figures S3 and S4). This species may aggregate in small chambers or enclosures where the air flow is relatively limited. The 5 SaV-infected specimens were all captured in a place called Tai Lam – Shek Kong located next to a major country park of Hong Kong, and this roosting site shares similar ecological characteristic with other sampled roosting sites. Due to the extremely high human population density in Hong Kong, direct contact between humans and bats is relatively frequent. Fortunately, no local case of bat zoonosis has ever been reported [36]. The relatively large genetic distance between the present bat SaV and other mammalian SaVs suggests that the zoonotic risk posed by this virus is likely to be low, though this should be confirmed with further *in vitro* and *in vivo* studies.

There are two main limitations in the current study. First and foremost, clinical information on the sampled bats is limited to the brief period of captivity needed for sample collection, which is unlikely to reflect the disease association of the virus accurately. In other words, the scope of the study is limited to surveillance of viral diversity and possible discovery of new viruses. Secondly, the number of samples for the novel virus is quite small, despite the use of specific PCR primers for screening and the relatively large number of samples collected. Thus, we were unable to draw conclusions on the seasonality of its detection or its host specificity. To address these limitations, long-term follow-up studies would be required to identify sufficient positive samples with associated clinical data. Increasing the scale of surveillance would also help, though there are practical geographical and logistic constraints in our locality.

In conclusion, we identified a novel bat SaV with several genomic features and properties that set it apart from other members of the genus *Sapovirus*. Phylogenetic analysis suggests that its ancestral lineage had diverged early from the other SaVs and evolved under different conditions. Further discovery and characterization of additional strains would enhance our understanding of the evolutionary history of the SaVs and other caliciviruses.

## Materials and Methods

### Surveillance and sample collection

The study was approved by the Department of Agriculture, Fisheries and Conservation, HKSAR; and Committee on the Use of Live Animals in Teaching and Research, The University of Hong Kong. Bats from 14 different locations in rural areas of Hong Kong, including water tunnels, closed mines, sea caves and forested areas, were captured over a 36-month period. Anal swabs were collected by an experienced veterinary surgeon, and kept in viral transport medium at 4°C before processing.

### RNA extraction

Viral RNA was extracted from the anal swabs using a QIAamp Viral RNA mini kit (Qiagen). The RNA was eluted into 50 µl RNase-free water and was used as the template for RT-PCR.

### RT-PCR of the RdRp region using conserved primers, and sequencing

Screening was performed by amplifying a 185 nt fragment in the RdRp region of the ORF1 gene of caliciviruses. Conserved degenerate primers (5′-GAYTAYTCNMRRTGGGAYTC-3′ and 5′- GGCATNCCNGAKGGNAYNCC -3′) were designed from the multiple sequence alignment of the available calicivirus gene sequences in NCBI GenBank. First-strand cDNA synthesis was performed using SuperScript III kit (Invitrogen) according to manufacturer's instructions. The PCR mixture (25 µl) contained cDNA, PCR buffer (10 mM Tris/HCl pH 8.3, 50 mM KCl, 2 mM MgCl_2_ and 0.01% gelatin), 200 µM of each dNTP and 1.0 U AmpliTaq Gold polymerase (Applied Biosystems). PCR cycling conditions were as follows: hot start at 94°C for 7 min, followed by 50 cycles of 94°C for 1 min, 50°C for 1 min and 72°C for 1 min with a final extension at 72°C for 10 min in an automated thermal cycler (Applied Biosystems). Standard precautions were taken to avoid PCR contamination and no false-positive signal was observed in the negative controls. The PCR products were gel-purified using a QIAquick gel extraction kit (Qiagen). Both strands of the PCR products were sequenced twice with an ABI Prism 3730xl DNA Analyser (Applied Biosystems), using the two PCR primers.

### RT-PCR screening of bat sapovirus using specific primers

Additional RT-PCR screening was performed on the same samples using specific primers designed from the RdRp nucleotide sequences of bat SaVs obtained from previous rounds of RT-PCR and sequencing, as RT-PCR screening with specific primers usually offers higher sensitivity than a comparable screening with consensus degenerate primers. Sequences of the specific primers are as follows: forward primer 5′- CACAATGCAGCCAGCCA-3′ and reverse primer 5′- GGTGCGCGTGGTGAACAC-3′. PCR cycling conditions were as follows: hot start at 94°C for 7 min, followed by 50 cycles of 94°C for 1 min, 52°C for 1 min and 72°C for 1 min with a final extension at 72°C for 10 min in an automated thermal cycler (Applied Biosystems). Standard precautions were taken to avoid PCR contamination and no false-positive signal was observed in the negative controls. PCR product purification and sequencing were performed as above.

### Cloning of PCR product and sequencing

Purified PCR products were cloned into a pCR2.1-TOPO vector (Invitrogen) according to manufacturer's instructions. The vector was then used to transform the competent Escherichia coli strain DH5α by electroporation. Positive transformants were identified by blue–white screening, and eight colonies were selected for DNA sequencing of the construct using the M13 forward and reverse primers according to the manufacturer's instructions. Sequencing reactions were performed as described above.

### Genome sequencing

Viral genome sequences were obtained using strategies we had previously used for other RNA viruses [50]–[52]. RNA extraction and cDNA generation were performed as described above. PCR primers were designed by targeting conserved regions, which were identified from the multiple alignment of genomes of related SaVs, as primer-binding sites. Additional primers for subsequent rounds of PCR were designed based on the results of earlier rounds of genome sequencing. The complete set of primer sequences is available from the authors upon request. The 5′ and 3′ ends of the viral genomes were sequenced following amplification of the segments by rapid amplification of cDNA ends, which was performed using the SMARTer RACE cDNA Amplification kit (Clontech) according to the manufacturer's instructions.

### Phylogenetic and genome analysis

ORFs were located using the ORF Finder tool at NCBI (http://www.ncbi.nlm.nih.gov/projects/gorf/). Annotation of the predicted proteins was performed by BLAST sequence similarity search against annotations in the NCBI RefSeq database. Multiple sequence alignments were constructed using MUSCLE version 3.8.31 [53], and phylogenetic informative regions were extracted using BMGE [54]. Maximum-likelihood phylogenetic trees were constructed using PhyML version 3 [55], under the best-fit protein evolution model as selected by ProtTest 3 [56]. Branch support values were estimated by calculation of SH-like aLRT values [57]. Recombination detection was performed by analysing the translated sequences of ORF1 and ORF2 separately using the Recombination Analysis Tool [58].

### Codon usage and compositional bias analysis

The full-length ORF1 and ORF2 coding sequences were extracted from selected SaV genomes and concatenated for codon usage analysis (see Table 4 for the list of included genome sequences). Codon usage and summary statistic of codon usage bias (N_c_ and N_c_′) were calculated using the INCA package version 2.1 [59], where N_c_ is the effective number of codons in the coding regions of the genome [60], and N_c_′ is the effective number of codons adjusted for background nucleotide composition [61]. For CpG dinucleotide bias analysis, odds ratio of CpG and GpC dinucleotides and the CpG/GpC ratio were calculated as described in previous studies [40], [41]. Odds ratio of ≤0.78 indicates significant suppression of the dinucleotide, same as the interpretation criteria of previous studies. Symmetrized nucleotide frequencies and dinucleotide odds ratio were not considered in the present study, as SaV genomes consist of positive-sense ssRNA only. To investigate the possible effects of cytosine deamination, genomic GC skew, which is the ratio (G-C)/(G+C), was calculated for the SaV genomes. The strength of the GC skew had been suggested to correlate with the degree of cytosine deamination [41], [44], [62].

## Supporting Information

Figure S1 Geographical distribution of the bat specimens in the present study.
(TIF)

Figure S2 Neighbor-joining tree of partial RdRp nucleotide sequences. The tree was constructed based on the length of the nucleotide sequence in the RdRp region obtained from bat SaV/TLC72.
(TIF)

Figure S3 Photo showing *Hipposideros pomona* is in the drainage at Tai Lam – Shek Kong.
(TIF)

Figure S4 Photo showing *Hipposideros pomona* possesses a small nose leaf.
(TIF)

Table S1 Epidemiology of the tested bat specimens.
(DOC)

Table S2 Amino acid identity of the Bat SaV/TLC58/HK with representative caliciviruses of other genera.
(DOC)
