# Supplementary figures and images for: Discovery and Genomic Characterization of a Novel Bat Sapovirus with Unusual Genomic Features and Phylogenetic Position
Source: PLoS One. 2012 Apr 13;7(4):e34987. doi: 10.1371/journal.pone.0034987 (PMC3325917; doi:10.1371/journal.pone.0034987)

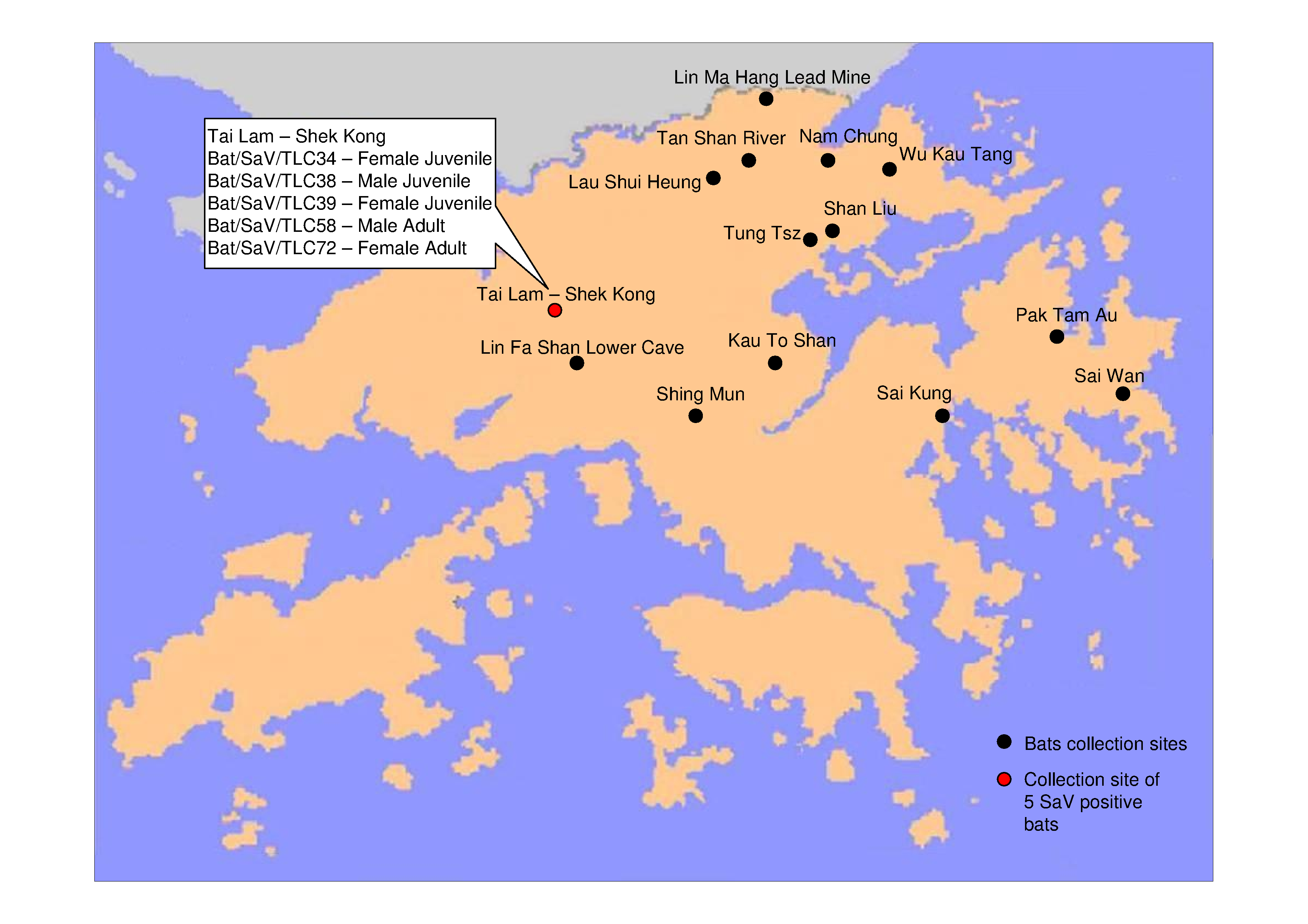

Supplement: Figure S1 — Geographical distribution of the bat specimens in the present study. (TIF) [file pone.0034987.s001.tif]

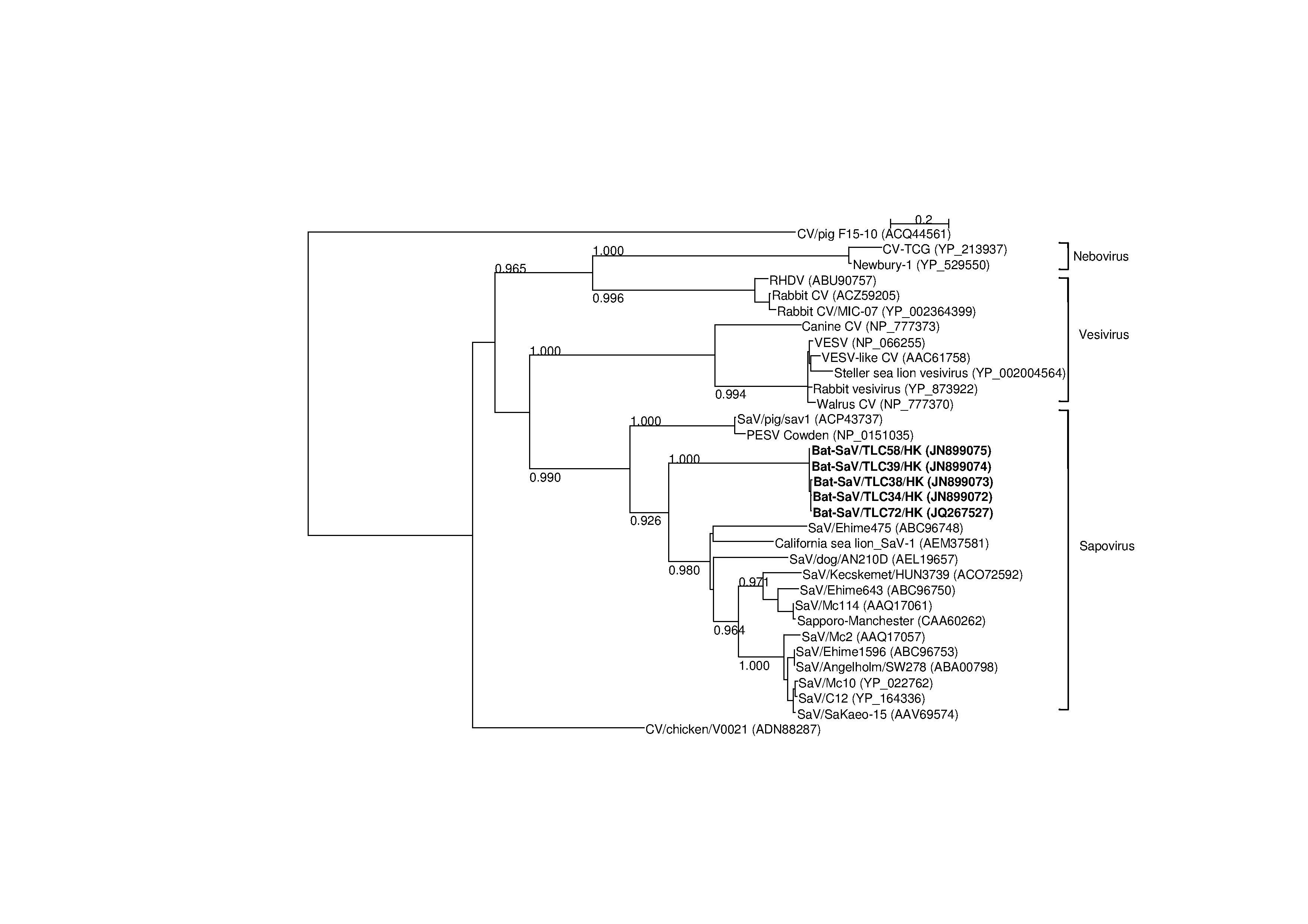

Supplement: Figure S2 — Neighbor-joining tree of partial RdRp nucleotide sequences. The tree was constructed based on the length of the nucleotide sequence in the RdRp region obtained from bat SaV/TLC72. (TIF) [file pone.0034987.s002.tif]

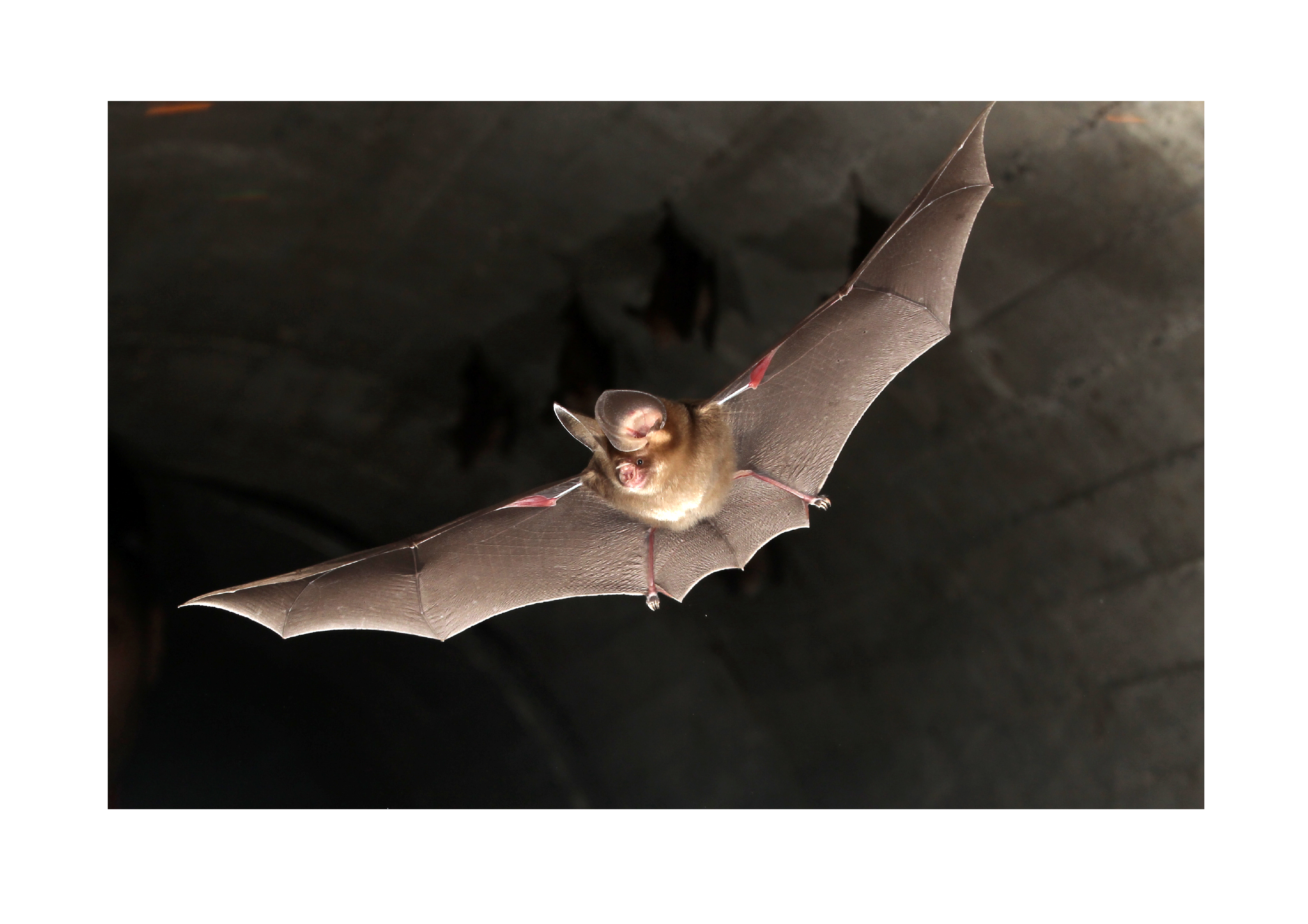

Supplement: Figure S3 — Photo showing Hipposideros pomona is in the drainage at Tai Lam – Shek Kong. (TIF) [file pone.0034987.s003.tif]

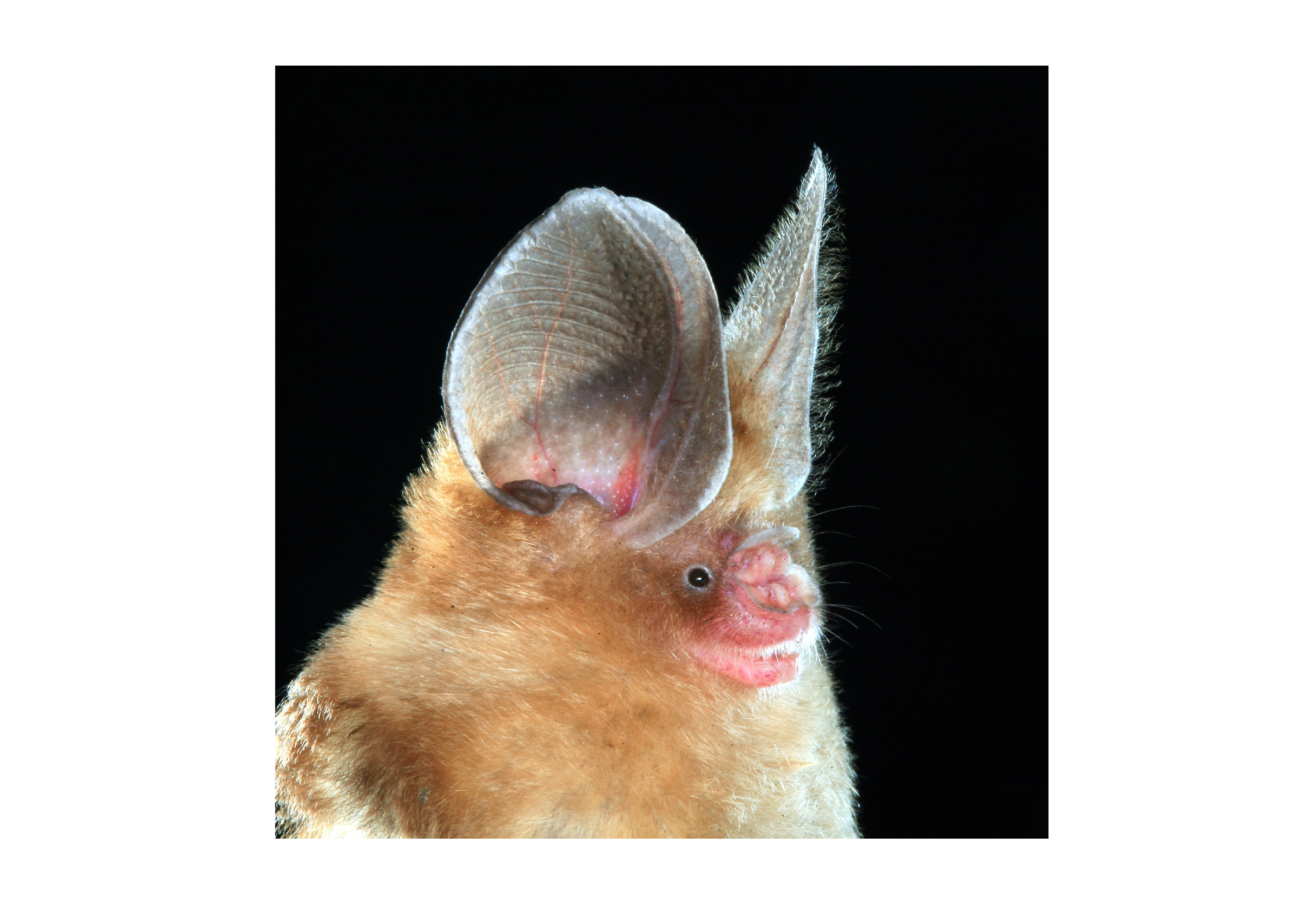

Supplement: Figure S4 — Photo showing Hipposideros pomona possesses a small nose leaf. (TIF) [file pone.0034987.s004.tif]
