# Supplementary material for: Discovery and Genomic Characterization of a Novel Bat Sapovirus with Unusual Genomic Features and Phylogenetic Position
Source: PLoS One. 2012 Apr 13;7(4):e34987. doi: 10.1371/journal.pone.0034987 (PMC3325917; doi:10.1371/journal.pone.0034987)
Supplement: Table S1 — Epidemiology of the tested bat specimens. (DOC) [file pone.0034987.s005.doc]

**Table S1.** Epidemiology of the tested bat specimens.

|  | **Number of animals screened by RT-PCR** | **Gender** | | **Stage** | | **Forearm range** | **Collection site** | **No. of collected** |
| --- | --- | --- | --- | --- | --- | --- | --- | --- |
|  |  | **Male** | **Female** | **Adult** | **Juvenile** |  |  |  |
| *Hipposideros armiger* | 14 | 7 (50%) | 7 (50%) | 8 (57%) | 6 (43%) | 86-95 | Shan Liu | 4 |
|  |  |  |  |  |  |  | Tai Lam – Shek Kong | 10 |
| *Myotis ricketti* | 103 | 45 (44%) | 58 (56%) | 100 (97%) | 3 (3%) | 45-85 | Nam Chung | 46 |
|  |  |  |  |  |  |  | Shan Liu | 1 |
|  |  |  |  |  |  |  | Tai Lam – Shek Kong | 56 |
| *Miniopterus pusillus* | 78 | 42 (54%) | 36 (46%) | 78 (100%) | 0 (0%) | 38-46 | Kau To Shan | 8 |
|  |  |  |  |  |  |  | Lin Ma Hang Lead Mine | 7 |
|  |  |  |  |  |  |  | Nam Chung | 22 |
|  |  |  |  |  |  |  | Tai Lam – Shek Kong | 41 |
| *Myotis chinensis* | 18 | 13 (72%) | 5 (28%) | 17 (94%) | 1 (6%) | 61-69 | Lin Ma Hang Lead Mine | 1 |
|  |  |  |  |  |  |  | Nam Chung | 3 |
|  |  |  |  |  |  |  | Tai Lam – Shek Kong | 14 |
| *Rhinolophus sinicus* | 65 | 18 (28%) | 47 (72%) | 44 (68%) | 21 (32%) | 40-48 | Lin Ma Hang Lead Mine | 1 |
|  |  |  |  |  |  |  | Shan Liu | 2 |
|  |  |  |  |  |  |  | Tai Lam – Shek Kong | 62 |
| *Tylonycteris pachypus* | 14 | 6 (43%) | 8 (57%) | 14 (100%) | 0 (0%) | 25-28 | Shing Mun | 13 |
|  |  |  |  |  |  |  | Tai Lam – Shek Kong | 1 |
| *Hipposideros pomona* | 321 | 137 (43%) | 184 (57%) | 292 (91%) | 29 (9%) | 39-47 | Kau To Shan | 15 |
|  |  |  |  |  |  |  | Lau Shui Heung | 143 |
|  |  |  |  |  |  |  | Lin Fa Shan Lower Cave | 20 |
|  |  |  |  |  |  |  | Nam Chung | 20 |
|  |  |  |  |  |  |  | Pak Tam Au | 10 |
|  |  |  |  |  |  |  | Sai Kung | 2 |
|  |  |  |  |  |  |  | Sai Wan | 1 |
|  |  |  |  |  |  |  | Shan Liu | 9 |
|  |  |  |  |  |  |  | Tai Lam – Shek Kong | 98 |
|  |  |  |  |  |  |  | Tung Tsz | 3 |
| *Pipistrellus abramus* | 9 | 1 (11%) | 8 (89%) | 5 (55%) | 4 (45%) | 30-33 | Shing Mun | 7 |
|  |  |  |  |  |  |  | Tan Shan River | 1 |
|  |  |  |  |  |  |  | Wu Kau Tang | 1 |
| *Miniopterus schreibersii* | 84 | 37 (44%) | 47 (56%) | 84 (100%) | 0 (0%) | 42-53 | Kau To Shan | 5 |
|  |  |  |  |  |  |  | Lin Ma Hang Lead Mine | 57 |
|  |  |  |  |  |  |  | Shan Liu | 11 |
|  |  |  |  |  |  |  | Nam Chung | 11 |
| *Nyctalus noctula* | 1 | 1 (100%) | 0 (0%) | 1 (100%) | 0 (0%) | 53 | Tan Shan River | 1 |
| *Pipistrellus* sp. | 2 | 1 (50%) | 1 (50%) | 2 (100%) | 0 (0%) | 38-40 | Wu Kau Tang | 2 |
| *Scotophilus kuhlii* | 1 | 1 (100%) | 0 (0%) | 1 (100%) | 0 (0%) | 53 | Tan Shan River | 1 |
| *Rhinolophus affinis* | 9 | 7 (78%) | 2 (22%) | 9 (100%) | 0 (0%) | 49-53 | Nam Chung | 8 |
|  |  |  |  |  |  |  | Wu Kau Tang | 1 |
| *Rhinolophus pusillus* | 9 | 6 (67%) | 3 (33%) | 9 (100%) | 0 (0%) | 33-40 | Kau To Shan | 7 |
|  |  |  |  |  |  |  | Lin Ma Hang Lead Mine | 1 |
|  |  |  |  |  |  |  | Nam Chung | 1 |
