# Supplementary material for: Discovery and Genomic Characterization of a Novel Bat Sapovirus with Unusual Genomic Features and Phylogenetic Position
Source: PLoS One. 2012 Apr 13;7(4):e34987. doi: 10.1371/journal.pone.0034987 (PMC3325917; doi:10.1371/journal.pone.0034987)
Supplement: Table S2 — Amino acid identity of the Bat SaV/TLC58/HK with representative caliciviruses of other genera. (DOC) [file pone.0034987.s006.doc]

**Table S2.** Amino acid identity of the Bat SaV/TLC58/HK with representative caliciviruses of other genera

| **Genus** | **Virus** | **Accession no.** | **Amino acid % Identity** | | | |
| --- | --- | --- | --- | --- | --- | --- |
|  |  |  | **ORF1 - NS** | **Pro-Pol** | **VP1** | **VP2** |
| Sapovirus | Manchester | X86560 | 35.9 | 47.6 | 36.8 | 17.4 |
|  | PEC | AF182760 | 36.1 | 46.3 | 37.4 | 19.3 |
| Norovirus | Jena | AJ011099 | 16.5 | 23.9 | 14.9 | 9.3 |
|  | Norwalk | M87661 | 15.6 | 23.3 | 16.2 | 12.3 |
|  | Southampton | L07418 | 16.5 | 23.8 | 16.9 | 11.9 |
|  | Hawaii | U07611 | 16.3 | 22.7 | 15.8 | 8.8 |
|  | Lordsdale | X86557 | 16.1 | 22.7 | 16.8 | 9.3 |
| Vesivirus | VESV-A48 | AF181082 | 22.8 | 29.0 | 20.0 | 5.7 |
|  | SMSV-1 | AF181081 | 22.7 | 28.6 | 19.6 | 5.7 |
|  | PAN-1 | AF091736 | 22.5 | 27.8 | 20.1 | 4.8 |
|  | WCV | AF321298 | 22.5 | 28.5 | 20.0 | 4.8 |
|  | FCV-F4 | D31836 | 22.4 | 27.7 | 19.5 | 4.8 |
| Lagovirus | EBHSV | Z69620 | 21.7 | 27.8 | 22.8 | 7.0 |
|  | RHDV | AF258618 | 21.6 | 27.9 | 23.4 | 7.0 |
| Nebovirus | BEC-NB | AY082891 | 22.0 | 32.1 | 21.9 | 10.0 |

For Sapovirus, lagoviru and nebovirus, the ORF1-NS polyprotein does not include the capsid protein VP1 encoded by ORF1

VESV: Vesicular exanthema of swine virus; SMSV: San Miguel sea lion virus; PAN: Primate calicivirus; WCV: Walrus calicivirus; FCV: Feline calicivirus; EBHSV: European brown hare syndrome virus; RHDV: Rabbit hemorrhagic disease virus; BEC: Bovine enteropathogenic calicivirus
